# Serial MRI Features of Canine GM1 Gangliosidosis: A Possible Imaging Biomarker for Diagnosis and Progression of the Disease

**DOI:** 10.1100/2012/250197

**Published:** 2012-03-12

**Authors:** Daisuke Hasegawa, Osamu Yamato, Yuya Nakamoto, Tsuyoshi Ozawa, Akira Yabuki, Kazuhito Itamoto, Takayuki Kuwabara, Michio Fujita, Kimimasa Takahashi, Shunta Mizoguchi, Hiromitsu Orima

**Affiliations:** ^1^Division of Veterinary Radiology, Department of Veterinary Science, Nippon Veterinary and Life Science University, Tokyo 180-8602, Japan; ^2^Laboratory of Clinical Pathology, Department of Veterinary Medicine, Kagoshima University, Kagoshima 890-0065, Japan; ^3^Kyoto Animal Referral Medical Center, Kyoto 613-0036, Japan; ^4^Animal Medical Center, Yamaguchi University, Yamaguchi 735-8515, Japan; ^5^Division of Veterinary Pathology, Department of Veterinary Science, Nippon Veterinary and Life Science University, Tokyo 180-8602, Japan

## Abstract

GM1 gangliosidosis is a fatal neurodegenerative lysosomal storage disease caused by an autosomal recessively inherited deficiency of **β**-galactosidase activity. Effective therapies need to be developed to treat the disease. In Shiba Inu dogs, one of the canine GM1 gangliosidosis models, neurological signs of the disease, including ataxia, start at approximately 5 months of age and progress until the terminal stage at 12 to 15 months of age. In the present study, serial MR images were taken of an affected dog from a model colony of GM1 gangliosidosis and 4 sporadic clinical cases demonstrating the same mutation in order to characterize the MRI features of this canine GM1 gangliosidosis. By 2 months of age at the latest and persisting until the terminal stage of the disease, the MR findings consistently displayed diffuse hyperintensity in the white matter of the entire cerebrum on T2-weighted images. In addition, brain atrophy manifested at 9 months of age and progressed thereafter. Although a definitive diagnosis depends on biochemical and genetic analyses, these MR characteristics could serve as a diagnostic marker in suspect animals with or without neurological signs. Furthermore, serial changes in MR images could be used as a biomarker to noninvasively monitor the efficacy of newly developed therapeutic strategies.

## 1. Introduction

GM1 gangliosidosis is a lysosomal storage disease caused by autosomal recessively inherited deficiency of *β*-galactosidase enzyme activity due to the mutations of *GLB1* gene [2]. The major clinical signs are progressive motor and psychointellectual disorders, visual defects, and premature death. At present, only symptomatic therapy is available, even for human patients. In animals, GM1 gangliosidosis has been reported in dogs, cats, calves, and sheep. In dogs, the disease has been detected in mixed-breed beagles [3], English Springer Spaniels (ESSs) [4], Portuguese water dogs (PWDs) [5], Alaskan huskies [6], Shiba Inu dogs (SIDs) [7], and a mixed-breed dog [8]. The molecular defects that cause the disease have been identified in PWDs [9], Alaskan huskies [10], and SIDs [11]. Therefore, these molecularly defined canine models are expected to provide versatile *in vivo* systems for testing newly developed therapeutic strategies such as gene and regeneration therapies.

 The clinical features of GM1 gangliosidosis in SIDs have been described in detail in several reports (Table 1) [1, 7, 12–15]. These clinical features in SIDs are similar to those of the late infantile/juvenile form (type 2) of human GM1 gangliosidosis [13]. This specific clinical information makes it possible for veterinary practitioners to more easily recognize sick dogs demonstrating the signs of GM1 gangliosidosis, although a definitive diagnosis depends on the demonstration of a deficient enzyme [16] and/or DNA mutation [11, 17, 18]. Furthermore, the clinical characteristics of this animal model are useful as a marker for the development and the testing of potential therapeutic methods for the human form of the disease [13].

 There have been several reports concerning MR images of human GM1 gangliosidosis [19–24], but only 2 reports examining MR findings in canine disease have been published [1, 19]. The canine reports describe the discrete MR features in an ESS and a PWD at 9 months of age [19] and 3 SIDs at 3–8 months of age [1]. However, serial MR changes from the preclinical to terminal periods should be determined in an animal model to establish MR biomarkers for both the reliable diagnosis and evaluation of the efficacy of newly developed therapeutic strategies. In the present study, we performed serial MR scanning from 2 to 11 months of age in one affected SID from a model colony. Furthermore, MR scans obtained between 3 and 10 months of age in 4 sporadic clinical cases possessing the same mutation were also analyzed to confirm the consistency of the serial change observed in the affected laboratory dog.

## 2. Materials and Methods

### 2.1. Laboratory Animals

One affected male SID from a previously established Japanese model colony [13] was subjected to serial MR scanning from the preclinical to terminal periods. MR scanning was performed once a month from 2 to 11 months of age (Table 2). This dog had received a commercial multivalent vaccine (Duramune MX8, Kyoritsu Seiyaku, Tokyo, Japan) at 6 and 9 weeks of age. The dog was isolated in a cage (72 cm wide, 124 cm long, and 81 cm high) and was allowed to exercise freely outside the cage for 2-3 hours a day. The room where the cage was placed was controlled at a temperature of 24–26°C and humidity of 40–60%, and the lighting was turned off during the night-time for 8–10 hours. The dog was fed commercial dog food twice per day and allowed to drink water *ad libitum* while it was capable of eating and drinking independently, that is, until approximately 8 months of age. After the dog developed abasia and/or astasia, it received nursing care including hand feedings, infusions, and heat retention. General physical, neurological, and hematological examinations were carried out once a month to periodically monitor the clinical status of the dog. At 11 months of age, the dog was euthanized humanely by an intravenous injection of pentobarbital sodium salt (approximately 125 mg/kg) due to the poor prognosis, and necropsy was performed immediately after the final MR scanning had been completed. As an age-matched control for the affected SID, a normal male Beagle was obtained from a colony of experimental dogs because the sizes of the body and cranium are very similar in the two breeds. This control dog was raised under the same conditions as the affected dog throughout the experimental period and underwent monthly MR scanning from 2 to 11 months of age (Table 2). The dogs were cared for and were used in the experiments in accordance with the guidelines for proper conduct of animal experiments issued by the Science Council of Japan [25]. All experimental procedures using experimental animals were performed in accordance with the guidelines regulating animal use at the Nippon Veterinary and Life Science University.

### 2.2. Clinical Cases

In the present study, MR images of 4 sporadic clinical cases (animals 1 to 4) possessing the same mutation as the laboratory dog were analyzed to confirm the consistency of the serial changes observed in the affected laboratory dog. The homozygous mutation was confirmed by a DNA test as previously reported [11]. The ages in months when these clinical cases underwent MR scanning are shown in Table 2. All animals underwent MR scanning a few months after the onset of disease (6 to 8 months of age) as a prediagnostic test prior to the DNA test. Only animal 1 incidentally underwent MR scanning before the onset of disease (3 months of age) due to a vertebral problem unrelated to GM1 gangliosidosis. These diagnostic MR images of animals 1 to 3 were previously reported [1]. In animals 1 and 2, MR scanning in the later stages of disease after the establishment of diagnosis was performed for the research purposes with owner consent (Table 2). All animals followed a clinical course similar to that shown in Table 1.

### 2.3. MR Methods

MR scanning of 2 laboratory dogs was carried out with a 1.5 Tesla superconducting MR imaging system (Visart, Toshiba, Tokyo, Japan) using a human knee coil as a radiofrequency coil under general anesthesia. The anesthesia was induced by the intravenous injection of propofol (7.0 mg/kg body weight), and then the dogs were intubated and maintained by the inhalation of isoflurane (1.5–2.0%) and oxygen. The anesthetized dogs were held in a sternal position on the table with their head in the radiofrequency coil. During the anesthesia, the heart beat, temperature, and end-tidal CO_2_ were monitored, and the respiration rate (8–12/min) was controlled by a ventilator. The scanning conditions were T2-weighted (T2W, fast-spin echo, TR/TE = 4000/100, matrix = 256 × 256, number of acquisition = 2), with fluid-attenuated inversion recovery (FLAIR, fast-spin echo, TR/TE/TI = 8000/120/2000, matrix = 256 × 256, number of acquisitions = 2), and T1-weighted (T1W, spin echo, TR/TE = 410/15, matrix = 160 × 256, number of acquisitions = 3) with and without intravenous gadodiamide contrast agent (0.2 mmol/kg, Omniscan, Daiichi, Tokyo, Japan). All images were obtained on a transverse plane (field of view 12 × 12 cm, slice thickness 2.5 mm, slice gap 0.5 mm) from the olfactory lobe to the caudal medulla oblongata. MR scanning of animals 1, 2, and 4 was carried out with a 0.3 Tesla MR imaging system with a permanent magnet (Airis-II Comfort, Hitachi, Tokyo, Japan) using a human knee coil as the radiofrequency coil. T2W, FLAIR, T1W, and gadodiamide contrast-T1W images were obtained in the transverse plane (slice thickness 3.5–4.0 mm, slice gap 0.5–1.0 mm), and some images were obtained in the sagittal and/or dorsal planes. Animal 3 was scanned with a 0.5 Tesla permanent magnet MR imaging system (Aperto Inspire, Hitachi). For MR scanning, these clinical cases were also anesthetized and monitored by employing almost the same protocol as that used for laboratory dogs. 

### 2.4. Imaging Analysis

All the obtained images were evaluated by 2 veterinary neuroradiologists (D. Hasegawa and H. Orima), and the findings were recorded. In the 4 dogs with serial imaging data (2 laboratory dogs and animals 1 and 2), the regions of interests (ROIs) were produced along the outline of the brain and ventricular systems, and the volume of brain parenchyma was measured as the area (cm^2^) of each ROI on the transverse FLAIR images from the first image displaying the lateral ventricles to the last image displaying the 4th ventricle. The area (cm^2^) of brain parenchyma was calculated by subtracting the ROI of the ventricular system from the ROI of the brain outline and then multiplying by slice thickness and slice gap to obtain the volume of brain parenchyma (cm^3^) in each slice. The total volume of brain parenchyma (cm^3^) was calculated by summing the volume of brain parenchyma in each imaging series. In addition, the interthalamic adhesion thickness (mm) that has been reported as an indicator of canine brain atrophy [26] was measured in each series. Brain volume and the interthalamic adhesion thickness measurements were consistently performed by one author (D. Hasegawa) using the same criterion.

### 2.5. Histopathology

The brain and other visceral organs of the affected laboratory dog that was euthanized at 11 months of age were presented for histological investigation. All organs were evaluated using hematoxylin and eosin stain. In addition, the brain sample was stained immunohistochemically using polyclonal rabbit anti-human myelin basic protein (MBP) antibody (DakoCytomation, Glostrup, Denmark) as a primary antibody followed by a secondary biotinylated goat anti-rabbit IgG antibody (Vector Laboratories, Burlingame, Calif, USA). Immunoreactivity was detected using a 3,3′-diaminobenzidine system (Merck, Darmstadt, Germany) after treatment with peroxidase-conjugate streptavidin (DakoCytomation). For this immunohistochemical staining, brain samples were obtained as a positive control from a 1-year-old Beagle that died of a cardiac disease and a 2-year-old mixed-breed dog that died of a bladder rupture.

## 3. Results

### 3.1. MR Findings

**Figure 1:**
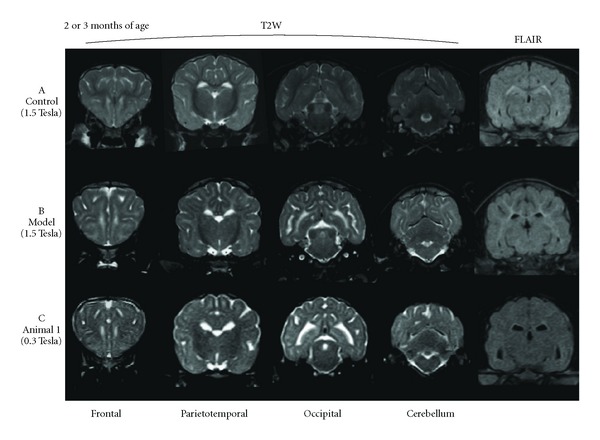
Various transverse sections of T2W and FLAIR images at 2 (A: age-matched control dog (control) and B: affected laboratory dog (model)) or 3 months of age (C: clinical case 1 (animal 1)). T2W hyperintensity in the subcortical white matter of the whole cerebrum was observed in affected dogs (B, C), but almost nonexistent in the control dog (A). T2W hyperintensity was more clearly recognized in B, which was obtained by a 1.5 Tesla system, than in C, which was obtained by a 0.3 Tesla system. In addition, subcortical hyperintensity was difficult to detect on the FLAIR images obtained by a 0.3 Tesla system.

**Figure 2:**
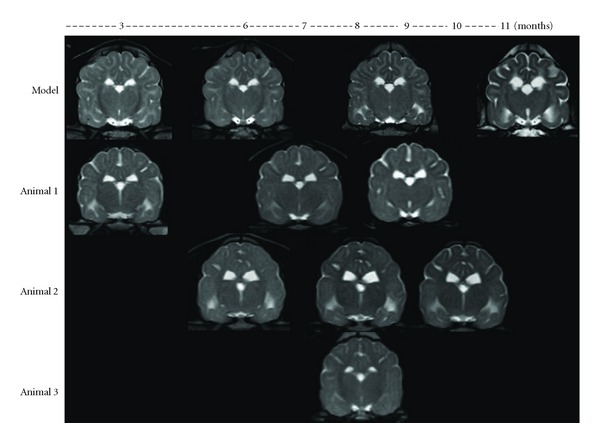
Serial changes in affected dogs (transverse T2W images at the thalamus/3rd ventricle level). Subcortical T2W hyperintensity was consistently observed at any timepoint. T2W hypointensity in the internal capsule was also recognized in the affected laboratory animal (model). Atrophic changes, which were indicated by ventricular enlargement and well-demarcated sulci, were observed in all affected dogs (model and animals 1–3) from 8 to 9 months of age.

**Figure 3:**
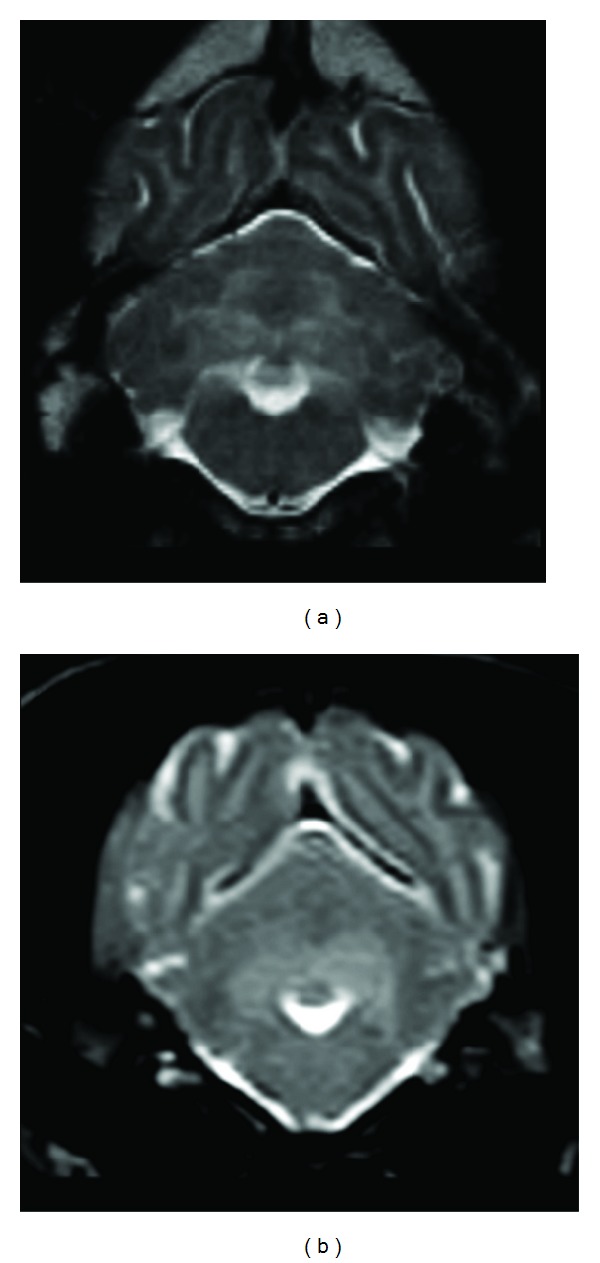
T2W hyperintensity in the white matter of the cerebellum (transverse image at the 4th ventricle level). (a) An affected laboratory dog at 9 months of age. (b) Clinical case (animal 1) at 9 months of age.

T2W and FLAIR hyperintensity in the subcortical white matter of the whole cerebrum was consistently observed on all images of the dogs affected with GM1 gangliosidosis regardless of timepoint (Figures 1(B), 1(C), and 2). Although this finding was discovered using multiple MR imaging systems that ranged from 0.3 to 1.5 Tesla, the images obtained with a 1.5-Tesla system more clearly demonstrated this abnormal finding (Figure 1(B)), while the FLAIR images obtained with a 0.3-Tesla system were not as clear (Figure 1(C)). In addition, there is an indication that this finding became more distinguishable with progression due to aging regardless of field strength (Figure 2). T2W hyperintensity in the white matter of the cerebellum was also observed from 7 months of age in the affected laboratory dog and in animal 1 at 9 months of age (Figure 3). However, this cerebellar T2W hyperintensity was not recognized in other clinical cases (data not shown).

**Figure 4:**
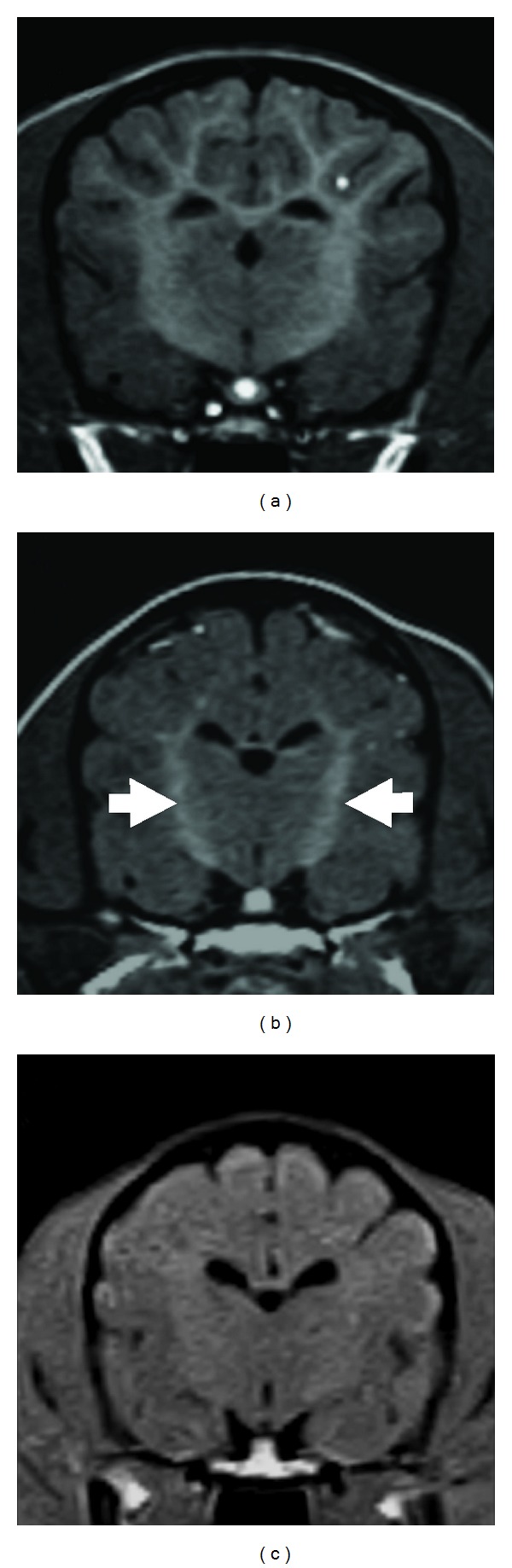
Transverse T1W images at the level of the thalamus/3rd ventricle. (a) An age-matched control dog at 2 months of age, (b) an affected laboratory dog at 2 months of age, (c) clinical case (animal 1) at 3 months of age. Symmetrical T1W hyperintensity was found in the internal capsule (arrows) in the affected laboratory dog (b), whereas there was no apparent hyperintensity in animal 1 (c). However, this internal capsule T1W intensity in the affected dog was not higher than that observed in the control dog (a).

 The affected laboratory dog presented symmetrical T1W hyperintensity and T2W hypointensity in the internal capsule from 2 months of age (Figures 1(B) and 4(b)). Thereafter, this T1W hyperintensity decreased gradually and resolved at 6 months of age, but the T2W hypointensity in the same region persisted until 11 months of age (Figure 2). This finding was not detected in any images of clinical cases, which were investigated using a lower field strength.

 On the serial MR images obtained from 3 dogs, that is, the affected laboratory dog and animals 1 and 2, ventricular enlargement and well-demarcated sulci were observed by 8 months of age (Figure 2), suggesting atrophic change.

 In the age-matched control dog, mild T2W hyperintensity was observed at the boundary between the cortex and medulla in the cerebrum, especially the frontal lobe, and in the margin of the lateral ventricles at 2 months of age (Figure 1(A)). However, this finding was almost resolved at 3 months of age and was not visible at all by 4 months of age. The other abnormal findings were not observed on either sequential images or at any timepoint.

### 3.2. Brain Volumetry and Interthalamic Adhesion Thickness

**Figure 5:**
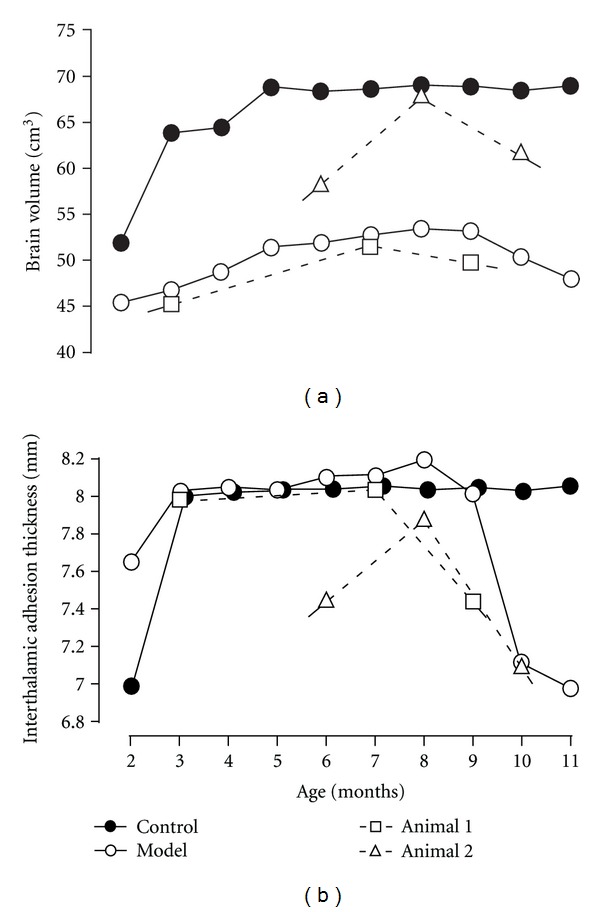
Changes in brain volumetry (a) and interthalamic adhesion thickness (b). Brain volume of the control dog (control) reached a steady volume at 3–5 months of age, whereas that of the affected dog (model) increased gradually until 8 months and then started to decrease. Similar changes were also found in interthalamic adhesion thickness.

The changes in brain volumes in 3 dogs, that is, the affected laboratory dog and animals 1 and 2, are shown in Figure 5(a). In the affected laboratory dog, the brain volume continued to increase until 8 months of age but started to decrease at 9 months of age and continued to decrease until the terminal stage. The interthalamic adhesion thickness, which is an indicator of canine brain atrophy, changed in the same way as the transition of the brain volume (Figure 5(b)). The changes in brain volume and interthalamic adhesion thickness in animals 1 and 2 were similar to those of the affected laboratory dog. However, the brain volume of the age-matched control dog increased rapidly until 3–5 months of age and then remained constant. Changes in the interthalamic adhesion thickness in this control dog were similar to those in the brain volume of this dog.

### 3.3. Histological Findings

**Figure 6:**
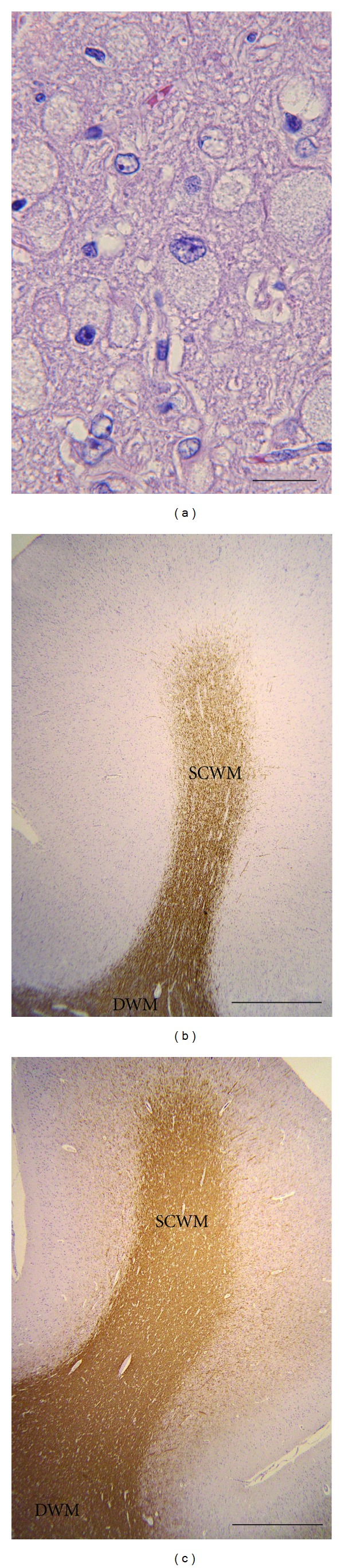
Histological findings in the affected laboratory dog. (a) High magnification of hematoxylin and eosin stain. Markedly swollen neurons filled with the storage materials were found throughout the central nervous system. Bar = 50 *μ*m. (b) Anti-MBP immunohistochemistry of the cerebrum in an 11-month-old affected dog and (c) a 2-year-old control mixed-breed dog. Poor myelination was particularly detected in the subcortical white matter in the affected dog compared with that in the control dogs. SCWM: subcortical white matter, DWH: deep white matter. Bar = 1 mm.

At necropsy, gross pathology of the affected laboratory dog revealed atrophic gyri and demarcated sulci. Histologically, the neurons were swollen like balloons throughout the central nervous system and filled with storage materials (Figure 6(a)). Reactive astrocytosis was observed in the neuropil. The development of the white matter appeared poor throughout the central nervous system, and vacuolation was found in some regions. Abnormal myelination, that is, dysmyelination or demyelination, was detected by a weak reaction to an anti-MBP antibody (Figure 6(b)) compared with that in positive control specimens (Figure 6(c)) and that of the subcortical white matter was more severe than those observed in other regions.

## 4. Discussion

The characteristic MR findings in SIDs with GM1 gangliosidosis were identified. MR findings consistently indicated diffuse hyperintensity in the white matter of the entire cerebrum on T2W images from 2 months of age at the latest and persisting until the terminal stage. Brain atrophy manifested at 9 months of age and progressed thereafter. These MR characteristics could be used as a diagnostic and/or therapeutic biomarker for this canine GM1 gangliosidosis model.

 Human GM1 gangliosidosis is classified into 3 types: infantile form (type 1), late infantile/juvenile form (type 2), and adult/chronic form (type 3) [2]. In canine GM1 gangliosidosis, it is thought that the disease in ESSs is similar to human type 1, while the disease in the other breeds, including SIDs, is similar to human type 2 [3, 5–7, 27]. Several reports describing MR findings in these 3 types of human diseases have been published. Reports describing types 1 and 2 indicate that symmetrical T2W hypointensity in the thalamus and diffuse T2W hyperintensity in the cerebral white matter result from delayed myelination [20, 21, 24]. In other reports, abnormal intensity on T2W images in the basal ganglia is observed in type 3 [28, 29]. To the best of our knowledge, however, there has been only one report in the English literature [19] and one report in the Japanese literature [1] concerning MR findings in canine GM1 gangliosidosis. The English report describes diffuse cerebral T2W hyperintensity in one ESS and one PWD at 9 months of age. The Japanese report describes diffuse cerebral T2W hyperintensity as the main finding in 3 SIDs in the early stage of the disease using relatively low field strength (0.3–0.5 Tesla). In the present study, we performed serial MR studies throughout the entire stage in one SID with GM1 gangliosidosis using higher field strength (1.5 Tesla) and demonstrated some novel findings that would be useful for an animal model.

 The most significant MR finding of the affected SIDs was T2W hyperintensity in the white matter of the entire cerebrum, which was consistently observed from the preclinical (at least 2 months of age) to terminal stages. This abnormality agrees with observations in human patients with types 1 and 2 of the disease, as well as those affected ESS and PWD at 9 months of age [19–22, 24]. Although it remains controversial, the cause of the T2W hyperintensity in the cerebral white matter has been attributed to primary hypoplasia of the myelin (hypomyelination) and/or delayed myelination (dysmyelination) [19, 22, 23, 30, 31] rather than secondary degeneration (demyelination) resulting from neuronal death due to the accumulation of storage materials [24, 32].

 In general, T1 and T2 values of the white matter are shortened by progression of myelination accompanied by brain development during the infantile period, and accordingly the white matter becomes hyperintensive on T1W imaging and hypointensive on T2W imaging [30, 33, 34]. Myelination of the cerebral white matter is completed by approximately 2-3 years of age in humans. In dogs, myelination may be completed at 3-4 months of age [35]. In the present study, the age-matched control dog presented diffuse T2W hyperintensity in the boundary between the gray and white matter of cerebrum, and this finding was resolved at 4 months of age. The change suggests that the myelination in the control dog was completed at approximately 4 months of age, similar to the literature reports [35]. This observation provided useful information to aid the diagnosis of GM1 gangliosidosis in animals 2 and 3, which underwent initial MRI at 6 months of age. In contrast, the differentiation between dysmyelination and infantile incomplete myelination was required in the affected laboratory dog at 2-3 months of age. However, as shown in Figure 1, the difference between dysmyelination in affected dogs and infantile incomplete myelination in a normal dog was obvious with respect to hyperintensity on T2W of the cerebral white matter. Therefore, these characteristics on MRI, especially T2W hyperintensity in the cerebral white matter, are a useful tool for antemortem and/or preclinical diagnoses of gangliosidosis in a suspected family or breed. However, the possibility of misreading T2W hyperintensity in the cerebral white matter remains when the images are obtained at lower field strengths. In the present study, it was sometimes difficult to recognize the diffuse T2W hyperintensity on images obtained using the 0.3-Tesla system.

 Hyperintensity on T2W of the cerebral white matter is not only specific for GM1 gangliosidosis but has also been found in other lysosomal storage diseases such as globoid cell leukodystrophy [31, 36] and GM2 gangliosidosis [37–42] in humans and animals. In gangliosidoses in domestic animals, diffuse T2W hyperintensity was observed in GM2 gangliosidosis variant 0 (Sandhoff disease) in Japanese domestic cats [41] and Toy Poodles [42], but not in the same disease in a Golden Retriever [43]. In canine Sandhoff's disease in these two breeds, the nucleus caudatus displayed bilateral T2W hyperintensity and T1W hypointensity [42, 43], which was not observed in SIDs with GM1 gangliosidosis in the present study. In contrast, there are some variations of MR findings even within human GM1 gangliosidosis. In a 7-month-old boy with the infantile form of GM1 gangliosidosis, dots and stripes with low signal intensity in diffuse and symmetrical T2W hyperintensity were seen in the cerebral white matter [31]. In an 18-month-old girl with GM1 gangliosidosis, T1W imaging presented persistent hyperintensity in the bilateral thalami, brainstem, and deep cerebellum [21]. In a 14-month-old girl with the late infantile form of the disease, MR examination indicated hyperintensity of the thalami on T1W images, while T2W images indicated decreased signal intensity of the thalami [20]. The report describing an adult type of the disease indicates that bilateral symmetrical putamen hyperintensity on T2W images is observed in more than 90% of the patients [29]. Therefore, the characteristics of MR findings in lysosomal diseases appear to depend on the molecular basis of the disease, not the mere presence of the disease itself.

 An additional finding, T2W hyperintensity in the deep white matter of cerebellum, was found in an affected laboratory dog and animal 1 in the late stage at 7 months of age. This finding was also observed on MR images of ESS and PWD at 9 months of age, which were recorded using relatively high field strength (1.0 Tesla) [19]. However, this finding was not observed clearly in the other animals in the present study. Therefore, these results suggest that the existence of increased T2W intensity in the cerebellar white matter depends on either individual differences or the detection ability of the MR imaging system. To the best of our knowledge, there has not been any report describing T2W hyperintensity of the cerebellar white matter in human GM1 gangliosidosis, but T2W hyperintensity has been reported in humans with Sandhoff's disease [44]. This change may result from progression of demyelination, increased storage materials, and astrocytosis, all of which were observed histopathologically in the affected laboratory dog.

 In the present study, T1W hyperintensity in the bilateral internal capsule was observed only in the affected laboratory dog in the early stages of the disease, although this finding was not described in the previous study of the affected ESS and PWD [19]. In human GM1 gangliosidosis, a similar T1W hyperintensity has been observed in the thalamus, which is thought to reflect developed myelination compared with the surrounding lesions [21]. Therefore, the T1W hyperintensity in the internal capsule seems to be produced by the progression of normal myelination in this region. The progression of myelination may exaggerate the T1W intensity against the surrounding T1W intensity due to delayed myelination that is shown as hyperintensity on T2W as describe above.

 Progressive brain atrophy was demonstrated using 2 quantitative methods for serial MR investigation. In human GM1 and GM2 gangliosidoses, progressive brain atrophy was observed in some patients who underwent serial MR imaging studies [21, 39, 40, 44]. Based on data from the affected laboratory dog and animals 1 and 2, brain atrophy seems to start at approximately 8-9 months of age and then progresses until the terminal stage. Interestingly, the brain volume of the affected dogs had a tendency to increase until approximately 8 months of age, while that of the age-matched control became constant at 3–5 months of age. These findings support the hypothesis that the volume of the gray matter is increased by the accumulation of storage materials in the lysosomes of cells until 8 months of age, and then cell death progresses dramatically resulting in progressive brain atrophy. In support of this hypothesis, we have reported previously that MBP in cerebrospinal fluid starts to increase significantly at 9 months of age in affected SIDs [45]. Together with the data from MRI and histopathological examination in the present study, the increased MBP may result from the breakdown of axonal myelin caused by the death of neurons.

 Large animal models such as dogs and cats are thought to be versatile for use in the development of new therapeutic strategies for human diseases. Canine and feline models have some disadvantages compared with rodent models; however, for example, not as many animals can be utilized for research. If it is possible to noninvasively evaluate the degree or extent of degeneration in the central nervous system using biomarkers obtained from living individuals, the number of animals necessary to determine the efficacy of potential therapeutic programs for GM1 gangliosidosis will be lower concurrently contributing to the welfare of experimental animals. These findings on serial MRI may provide a useful diagnostic and/or therapeutic biomarker for GM1 gangliosidosis as well as clinical features (Table 1) and previously reported cerebrospinal fluid biomarkers [45].

## Figures and Tables

**Table 1:** Summary of clinical features in Shiba Inu dogs with GM1 gangliosidosis.

Age (months)	Clinical features
Birth—5	No specific neurological signs, but abnormal cytoplasmic vacuoles in 30–50% of lymphocytes are always observed on blood smears. Ranula of the sublingual salivary gland with unknown etiology may be observed during this period or later.
5-6	Loss of balance; intermittent lameness; mild to moderate ataxia; dysmetria mainly hypermetria; head tremor; intention tremor.
7-8	Severe ataxia; loss of postural reactions; falling; exaggeratedly startled response to touch and sounds.
9-10	Atactic abasia; astasia; slight corneal clouding; visual disorder; muscle rigospasticity in limbs and crest; slight mental retardation.
11-12	Generalized muscle spasticity; tonic spasm; tendency to be lethargic; unresponsive to sounds; weight loss.
12–15	Generalized extensive rigor; lethargy; stuporous; death: median age at death is approximately 14 months, but one dog died at 18 months of age.

**Table 2:** Dogs and their age in months at the time of MR scanning.

Age (months)	2	3	4	5	6	7	8	9	10	11
Laboratory dog	D	D	D	D	D	D	D	D	D	D
Animal 1		D*				D*		D		
Animal 2					D*		D		D	
Animal 3							D*			
Animal 4						D				
Control	D	D	D	D	D	D	D	D	D	D

D: MR scanning was performed. *These MR images were reported previously as prediagnostic data in animals 1 to 3 [1].
